# Remembering to choose the future

**DOI:** 10.7554/eLife.49828

**Published:** 2019-08-14

**Authors:** Lesley K Fellows

**Affiliations:** 1 Department of Neurology and Neurosurgery, Montreal Neurological InstituteMcGill UniversityMontrealCanada

**Keywords:** decision making, hippocampus, fMRI, amnesia, value, Human

## Abstract

A brain region known as the hippocampus is required when people assess different options before making a value-based choice.

**Related research article** Bakkour A, Palombo DJ, Zylberberg A, Kang YHR, Reid A, Verfaellie M, Shadlen MN, Shohamy D. 2019. The hippocampus supports deliberation during value-based decisions. *eLife* **8**:e46080. doi: 10.7554/eLife.46080

From the philosophers of ancient Greece to the self-help books of today, humans have long been interested in choice. Philosophers and ethicists have debated what goals we *ought* to choose for millennia, and for a century or more economists and psychologists have studied what goals we *will* choose. However, neuroscience has only recently begun to systematically address *how* we choose.

Whether we are pondering life-defining decisions about love, career or commitment to a cause, or simply picking which snacks to buy in the grocery store, it is still unclear what regions of the brain are involved in making choices, and what information those regions encode. In everyday language, we often talk about ‘value’ (or in economic terms, ‘utility’) as the driver of such decisions: we consider our options, and select the one with the highest value. Hundreds of functional MRI (or fMRI) studies in healthy humans have identified a consistent set of brain regions which seem to process signals associated with subjective values; this suggests that value is indeed a concept that has biological roots (Bartra et al., 2013). However, the nature of the information that contributes to the neural signals related to value remains a matter of debate (O'Doherty, 2014). In other words, it is not clear what we think about when we think about value.

In fact, scientists know far less about choices based on value than they do about perceptual decisions (such as assessing if a noisy array of moving dots is trending more to the left or to the right; Shadlen and Kiani, 2013). During perceptual choices, external information is repeatedly sampled and the neural representation of this evidence accumulates until a threshold is crossed and a decision is triggered. These tasks are associated with well-known behavioral phenomena – for instance, choices with less perceptual evidence take longer to resolve – which are captured by drift diffusion models (Ratcliff and McKoon, 2008).

It has been proposed that value-based decisions might occur in a similar way (Rangel et al., 2008). However, while it is obvious what knowledge is accumulating as a person gazes at a screen filled with moving dots, it is less clear what information might be sampled to support a decision based on value. Now, in eLife, Akram Bakkour of Columbia University and colleagues report that, at least in part, we may be thinking about past experiences (Bakkour et al., 2019).

Their work makes a strong case that value-based deliberation engages the hippocampus, a small structure within the brain that is involved in long-term memory. Although past experiences are a likely source of relevant information in value-based decisions, to date researchers have focused mostly on other regions of the brain such as the ventral prefrontal cortex and the striatum.

Bakkour et al. – who are based at Columbia and the Memory Disorders Research Center – first used fMRI to establish that activity in the hippocampus is greater for longer deliberations during value-based choice. They then harnessed the power of a lesion experiment to infer that the structure is necessary for such choices (Vaidya et al., 2019). Patients with hippocampal damage were slower to make decisions, and somewhat more variable in what they chose. These hippocampal effects were specific to value-based decisions. Deliberation time in a classic perceptual decision task did not relate to hippocampal signal, nor was it influenced by hippocampal damage. While perceptual decisions involve sampling external evidence, Bakkour et al. propose that deliberation during value-based choice requires sampling internal evidence. This includes – although is presumably not limited to – using the hippocampus to conjure up past experiences with similar options. Ultimately, these results will help to broaden the anatomical scope of decision neuroscience.

Studies have already shown that ‘attention’, while intuitive and attractive as a holistic concept, is in fact composed of dozens of distinct processes with definable characteristics that rely on different neural circuits. It is likely that ‘value’ will also require further decomposition. Armed with this knowledge, it may become possible to better understand how the brain carries out the important value-based decisions that define us as individuals and shape the directions of our societies.
